# Estimated Renal Metabolomics at Reperfusion Predicts One-Year Kidney Graft Function

**DOI:** 10.3390/metabo12010057

**Published:** 2022-01-10

**Authors:** Thomas Verissimo, Anna Faivre, Sebastian Sgardello, Maarten Naesens, Sophie de Seigneux, Gilles Criton, David Legouis

**Affiliations:** 1 Laboratory of Nephrology, Department of Medicine, University Hospitals of Geneva, 1205 Geneva, Switzerland; thomas.verissimo@unige.ch (T.V.); anna.faivre@unige.ch (A.F.); sophie.deseigneux@hcuge.ch (S.d.S.); 2 Department of Surgery, University Hospital of Geneva, 1205 Geneva, Switzerland; sgardello@outlook.com; 3 Service of Nephrology, University Hospitals of Leuven, 3000 Leuven, Belgium; maarten.naesens@uzleuven.be; 4 Service of Nephrology, Department of Internal Medicine Specialties, University Hospital of Geneva, 1205 Geneva, Switzerland; 5 Geneva School of Economics and Management, University of Geneva, 1205 Geneva, Switzerland; criton.gilles@yahoo.com; 6 Division of Intensive Care, Department of Acute Medicine, University hospital of Geneva, 1205 Geneva, Switzerland

**Keywords:** AKI (acute kidney injury), renal transplantation, machine learning, metabolomics

## Abstract

Renal transplantation is the gold-standard procedure for end-stage renal disease patients, improving quality of life and life expectancy. Despite continuous advancement in the management of post-transplant complications, progress is still needed to increase the graft lifespan. Early identification of patients at risk of rapid graft failure is critical to optimize their management and slow the progression of the disease. In 42 kidney grafts undergoing protocol biopsies at reperfusion, we estimated the renal metabolome from RNAseq data. The estimated metabolites’ abundance was further used to predict the renal function within the first year of transplantation through a random forest machine learning algorithm. Using repeated K-fold cross-validation we first built and then tuned our model on a training dataset. The optimal model accurately predicted the one-year eGFR, with an out-of-bag root mean square root error (RMSE) that was 11.8 ± 7.2 mL/min/1.73 m^2^. The performance was similar in the test dataset, with a RMSE of 12.2 ± 3.2 mL/min/1.73 m^2^. This model outperformed classic statistical models. Reperfusion renal metabolome may be used to predict renal function one year after allograft kidney recipients.

## 1. Introduction

Chronic kidney disease (CKD) is defined as an alteration in renal structure or function during a period of at least 3 months [1]. CKD is a major global health burden [2], affecting about 10% of the world’s population. Many CKD patients progress to end-stage renal disease (ESRD) and 2.4 million people are currently receiving renal replacement therapy (RRT) [3,4], a number that is expected to rise dramatically by 2030 [4]. Renal transplantation remains the gold standard treatment for renal replacement therapy in ESRD patients [5,6], improving both quality of life and life expectancy [5,6,7]. However, while long-term renal graft survival has gradually improved during the last decade [7], progress is still needed in order to meet the demand created by increasing numbers of waitlisted patients [8]. Therefore, early identification of patients at risk of poor renal graft survival is critical in optimizing their management [9]. Renal function within the first year of transplantation has been proven to be strongly associated with long-term graft survival [10,11] and thus may be an ideal predictor of graft survival.

Kidneys are the organs with the second-highest metabolic rate [12], and several studies have recently highlighted the critical role played by renal metabolism in both acute kidney injury (AKI) and CKD [13,14,15,16,17,18]. Metabolomics, the comprehensive study of metabolites, is increasingly becoming a powerful tool in understanding renal pathophysiology and in the identification of new biomarkers [19]. In the field of renal transplantation, metabolomics studies have reported interesting results; however, they are mostly limited to short time points from transplantation [20,21,22,23,24,25], mostly focusing on delayed graft function, while later outcomes remain to be explored. 

Furthermore, collecting and analyzing metabolomics data in large cohorts is challenging. Machine Learning (ML) algorithms adopt a data-driven approach and are able to learn optimal solutions for the analysis of new data. Among the available algorithms, random forest (RF) has been widely used in the field of renal metabolomics [26,27,28,29]. This technique generates a multitude of decision trees that have different experiences of the problem. Once all the decision trees have been trained, RF makes its predictions by voting on all of its decision trees.

In this study, we took advantage of a recently developed method which is able to estimate metabolome abundance from RNAseq data [30]. This technique was applied on transcriptomes of kidney biopsies sampled at reperfusion. The estimated metabolites’ abundance was further used to predict the one-year allograft renal function via an RF machine learning algorithm.

## 2. Results

### 2.1. Description of the Cohort

Forty-two kidney allograft recipients, in which a renal biopsy was performed at reperfusion, were included. Bulk RNA-sequencing technology was then applied on those samples and the patients were followed-up on for one year [31]. Patients’ characteristics, split by the median of the one-year eGFR (51 mL/min/1.73 m^2^), are shown in Table 1. Patients with the highest one-year eGFR received a renal graft from a younger donor (39 versus 56 years) with a lower body mass index (24 versus 27 kg/m^2^). Other recorded characteristics did not significantly differ among groups.

**Figure 1:**
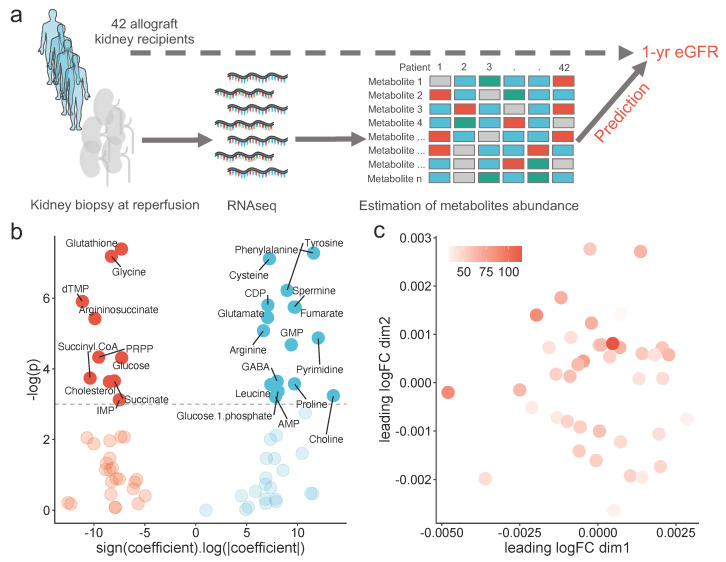
Global description of the study: (**a**) schematic representation of the study design, (**b**) volcano-plot showing the signed logarithm of the absolute ß coefficient extracting from a robust linear model using one-year eGFR as the dependent variable, and its associated *p*-value, for each estimated metabolite abundance, and (**c**) scatter plot showing the multidimensional scaling of the estimated metabolomics, colored by the one-year eGFR.

Using the renal transcriptomics data as an input, we used single-cell Flux Estimation Analysis (scFEA) to estimate the metabolites’ abundance within the renal graft at reperfusion [30]. An outline of the study design is displayed in Figure 1a. The resulting matrix was analyzed through multidimensional scaling and did not cluster patients according to their one-year eGFR (Figure 1b). For each estimated compound, we fitted a robust linear regression using the one-year eGFR as the dependent variable. The signed logarithm of the ß coefficient extracted from every model is plotted against its associated *p*-value in Figure 1c. A total of 26 estimated metabolites were significantly associated with one-year eGFR in a linear fashion, 16 positively and 10 negatively.

**Figure 2:**
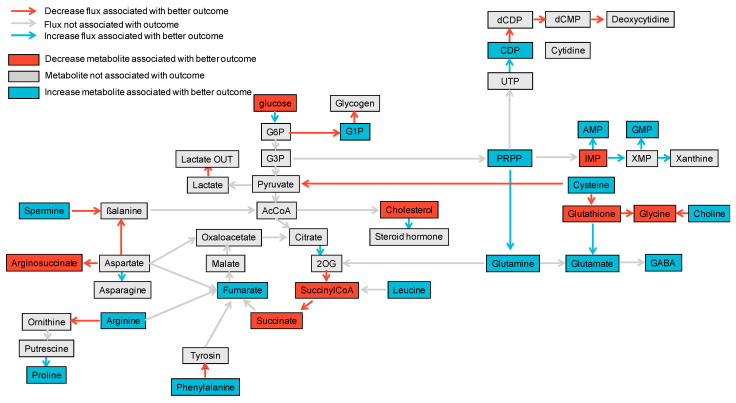
Comprehensive map linking estimation of fluxome and metabolites’ abundance with one-year eGFR. In blue are any positive associations between the metabolites’ estimated abundance or estimated reaction flux with one-year eGFR while in red are negative associations. Non-significant associations are shown in grey.

Altogether, we were able to build a comprehensive map displaying whether or not each metabolite or reaction flux, which was estimated at reperfusion, was associated with one-year eGFR (Figure 2, Supplementary Figure S1a,b).

### 2.2. Machine Learning on Estimated Metabolomic Predicts One-Year eGFR

Using the estimation of the metabolite’s abundance, we predicted the one-year eGFR. However, the input data presented many challenges. Firstly, analyses of the estimated metabolites’ distribution revealed the presence of outliers (Supplementary Figure S2a). Secondly, some of the estimated compounds displayed a nonlinear association with the one-year eGFR (Supplementary Figure S2b). Thirdly, some estimated metabolites were highly correlated with the 1-year eGFR, 14 of them having a Pearson coefficient above 0.8 (Supplementary Figure S2c). Lastly, the number of predictors (70) exceeded the number of patients (42), and the outcome measure displayed a skewed distribution (Supplementary Figure S2a).

To handle this data, we chose to perform an RF machine learning algorithm and followed a classic pipeline of analyses [32,33]. 

We first randomly split the whole dataset in a training and a test partition, using a 0.8:0.2 ratio. The distribution of one-year eGFR among the two sets of data is shown in Supplementary Figure S2d and Table 2.

We then trained an RF on the training dataset using a repeated K-fold cross-validation resampling method. A hyperparameter grid was used to tune the model, whose performance was iteratively assessed by the Root Mean Square Error (RMSE). The optimal model that minimizes the RMSE presented the following hyperparameters: 56 for mtry (the number of predictors randomly samples as candidate at each node split), 25 for ntree (the number of trees to grow) and 8 for the nodesize (the minimum size of terminal node) (Supplementary Figure S3a).

**Figure 3:**
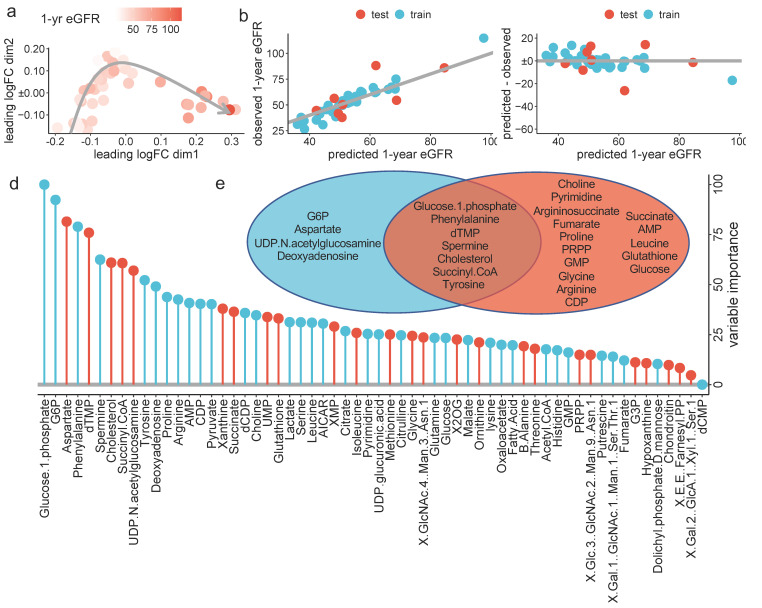
ML-based prediction of the one-year eGFR: (**a**) Multidimensional reduction plot showing the similarity of samples and colored by one-year eGFR, (**b**) scatter plot showing the observed (*y*-axis) predicted (*x*-axis) one-year eGFR, in the test (red) and training (blue) datasets. The grey line highlights the perfect relation, (**c**) Scatter plot showing the residuals for each predicted value, in the test (red) and training (blue) datasets. (**d**) Variable importance in the RF model for one-year eGFR prediction. Values are normalized to the most important variable, which was set to 100, and colored in blue if the variable is associated with a higher one-year eGFR and in red if associated with a lower one-year eGFR and (**e**) Venn diagram reporting the logical relation between the top 11 variable used in the RF model (blue) and the variables significantly associated with the one-year eGFR identified via robust linear regression (red), after exclusion of correlated variables (i.e., glutamate, cysteine, IMP and GABA).

By applying this model on the whole dataset, we were able to build a proximity matrix. Using Multidimensional Scaling (MDS), we could two-dimensionally represent the distances between samples. The dots were found to be ordered according to the one-year eGFR (Figure 3a). 

In the training dataset, the out-of-bag RMSE was 11.8 ± 7.2 mL/min/1.73 m^2^, similar to that obtained in the test dataset, which was 12.2 ± 3.2 mL/min/1.73 m^2^ (Table 3). Figure 3b,c shows the observed one-year eGFR and the residuals, respectively, plotted against the predicted one-year eGFR for both the training and test cohorts.

Lastly, we extracted the importance of every estimated metabolite for the one-year eGFR prediction from the optimal RF model. The output is shown in Figure 3d. We further colored the bars according to its positive or negative marginal effect on outcome, depending on the partial dependance plot (Supplementary Figure S3b). 

RF was able to capture nonlinear association between metabolites and one-year eGFR (Figure 3e and Supplementary Figure S3b).

### 2.3. ML Approach Outperforms Classic Statisical Method

We thus aimed at comparing the performance of the ML model trained on the estimated metabolome to a more classic approach, both in terms of the modeling technique and the set of predictors. To do this, we performed linear regression using the available biological and clinical variables as predictors, instead of estimated metabolites’ abundance.

**Figure 4:**
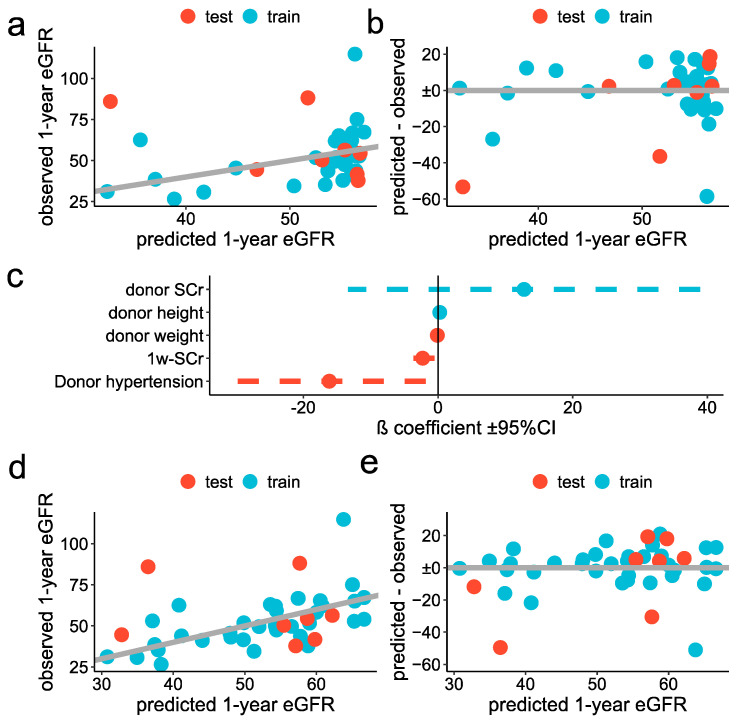
Classic statistical approach for prediction of the one-year eGFR: (**a**,**b**) Simple model: scatter plot showing the observed (*y*-axis) predicted (*x*-axis) one-year eGFR, in the test (red) and training (blue) datasets (**a**), scatter plot showing the residuals for each predicted value, in the test (red) and training (blue) datasets (**b**). (**c**–**e**) stepwise regression: forest plot showing the ß coefficient ±95% confidence interval of each variable selected in the optimal model (**d**), scatter plot showing the observed (*y*-axis) predicted (*x*-axis) one-year eGFR, in the test (red) and training (blue) datasets and (**e**) scatter plot showing the residuals for each predicted one-year eGFR, in the test (red) and training (blue) datasets.

As 1-week serum creatinine (SCr) is associated with long-term renal function [10,34], we started with a simple model using only 1-week SCr to predict one-year eGFR. This model displayed an RMSE of 13.3 ± 8.1 mL/min/1.73 m^2^ in the training dataset that dramatically increased in the training dataset (RMSE equal to 24.4 ± 7.5 mL/min/1.73 m^2^, Table 3), suggesting overfitting (Figure 4a,b).

As a second model, we adopted a more sophisticated technique. As missing data were present for three variables (donor serum creatinine, donor age and warm ischemia time), we first put in data using bagged tree imputation [35]. The distribution of those variables, before and after the imputation, is reported in Supplementary Figure S4. We thus filtered potential predictors by the significance levels of the univariable association with one-year eGFR (cutoff threshold set to 0.3) and took advantage of repeated K-fold cross-validation to find the optimal predictors’ combination, using stepwise selection. We trained the model by fixing the number of final predictors from 1 to the total number of available variables. The optimal model included 5 predictors, with their ß coefficients reported in Figure 4e. The RMSE was 12.7 ± 7.0 mL/min/1.73 m^2^ and 23.2 ± 6.2 mL/min/1.73 m^2^ in the training and test datasets, respectively (Figure 4d,e and Table 3). 

## 3. Discussion

In this study, we applied scFEA software to estimate reaction fluxes and abundance of metabolites in renal grafts, sampled at the time of transplant reperfusion. Those data were further used to (1) draw a comprehensive map of the association between estimated renal metabolism at reperfusion and one-year eGFR, and (2) to predict the renal graft function at one year. This outcome was selected as the renal graft function one year after transplantation has been largely identified as a major factor associated with graft survival [34,35,36,37,38,39,40,41]. Using multivariable analyses, two studies have also shown that estimated one-year GFR was the best predictor of long-term renal graft survival [10,11]. The one-year eGFR was part of a tool designed to predict the risk of graft loss within the first five years of transplant [42]. Finally, the eGFR at one year post kidney transplantation has been widely used in clinical trials as a surrogate endpoint for long-term renal graft outcomes [43].

We observed a positive association between the one-year eGFR and the estimated flux from glucose to glucose-6-phosphate (G6P), the first step of glycolysis. Glucose metabolism is modified during AKI [13,17], and glycolysis enhancement has been described as protective in AKI [44,45,46,47]. In this situation, glycolysis may ensure energy production, even at low levels, to provide the amounts of ATP necessary to maintain cell viability at the acute phase of the injury and to initiate the repair [12,47,48]. On the contrary, the estimated synthesis of glycogen from glucose-1-phosphate (G1P) was decreased in patients exhibiting a worse outcome. This is in line with recent studies reporting a deleterious effect of renal glycogen accumulation during glycogen storage disease type 1 [49,50]. Among metabolites associated with one-year eGFR, three (i.e., succinyl-CoA, succinate and fumarate) are part of the Tricarboxylic Acid (TCA) cycle. Numerous studies have shown that the mitochondria and TCA cycles are dysregulated at the renal level during AKI and CKD [16,17,50,51,52]. We noticed that the estimated succinate levels were linked to a worse outcome, while the opposite was observed for fumarate. In CKD patients, similar findings have been reported, where succinate and fumarate levels were negatively and positively associated with the eGFR, respectively [53]. In addition, we found the abundance of several amino acids (i.e., phenylalanine, leucine, glycine, proline, arginine) to be significantly linked to one-year eGFR, in accordance with reports from other groups in CKD patients and in animal models of CKD [54,55,56,57,58]. Lastly, spermine abundance was associated with a better outcome. This is in line with the improvement in renal outcome after AKI by spermidine supplementation [59] or the inhibition of catabolism by spermidine/spermine N1 acetyltransferase [60]. However, when considering CKD, both spermine and spermidine serum levels were increased in animals [61].

The analysis of metabolic data presents significant challenges, in particular due to the large amount of generated material, which includes many collinear variables and non-linear associations with phenotypic traits as well as the typically low number of samples [62]. In this context, a data-driven approach via a machine learning algorithm has gained prominence in recent years [63]. Among the available methods, RF is reported as an excellent regressor, with several advantages, including fast-speed, noise insensitivity and overfitting robustness [64]. RF combines two ML methods: bagging and random feature selection. Briefly, RF builds an ensemble of independent decision trees from multiple samplings of the original dataset. At each node of the tree, only one random selection of the available predictors is considered for the partition of the node, thus reducing the trees’ similarity grown from different samples. Once a sufficiently large forest of trees has been grown, the results are bagged in the usual way [65,66,67]. An RF algorithm also has the ability to measure the importance of each predictor, i.e., how it contributes to the prediction performance [29]. In our work, the optimal RF model was chosen to minimize the RMSE, in order to penalize large errors. However, it is interesting to note that the optimal model did not differ when using the R-squared or the mean absolute error metrics. We found the ML approach to largely outperform classic statistical approaches, which were characterized by a high degree of overfitting. Thus, our RF model displayed an R-squared of 0.6 and 0.5 in the training and test cohorts, respectively, meaning that 60 and 50% of the one-year eGFR variability is explained by the model. Interestingly, some metabolites that did not show any significant association with one-year eGFR displayed a high importance in the RF model that well captured their non-linear association with the outcome.

Our study has three main limitations. Firstly, the metabolites’ abundance has been estimated and not measured. Therefore, we cannot claim to obtain similar results using measured metabolomics profiles. However, in their original manuscript, the authors of the scFEA algorithm validated its prediction with several matched metabolomics data [30]. They also successfully applied their software in another study [68]. Secondly, we did not validate our prediction model on an external cohort. However, we trained our model using cross-validation and further assessed its performance in a test dataset not used during the model building. Thirdly, our sample size was limited, although the similar low RMSE calculated in both training and test datasets suggests good calibration without under or overfitting.

This work paves the way for further studies to develop clinical applications using, for example, urinary metabolites, which have been found to reflect the renal metabolite composition [69]. Ultimately this technique could be part of clinical trials, allowing for more research into the early and individualized management of renal graft recipients to extend transplant survival.

Our study is a proof of concept. Renal metabolomics may be used to predict, at a very early point in time, long-term renal graft survival. This approach, in combination with ML algorithms, seems much more accurate than the classic mixed clinical and biological approach. 

## 4. Materials and Methods 

### 4.1. Inclusion of Patients

Using a biobank of kidney transplant biopsies, we included allograft kidney recipients from the University Hospitals of Leuven according to the following criteria: (1) signed informed consent for Biobank Kidney Transplantation, (2) single kidney transplantation, (3) at least one year of follow-up, (4) availability of all material and (5) kidney transplantation performed between March 2012 and May 2015. Forty-two patients were included as previously described [31]. For each patient, the eGFR within the first year of transplantation was calculated with the CKD-EPI formula [70]. The study was conducted according to the guidelines of the Declaration of Helsinki, and approved by the Ethical Review Board of the University Hospitals of Leuven (S53364 and S59572). 

### 4.2. Estimation of Metabolomics

For each included patient, bulk-RNA sequencing technology was applied to kidney reperfusion biopsies as previously described [31]. Raw data are available at GEOi accession GSE126805. We performed normalization by the trimmed mean of M-value method (TMM) using the EdgeR package (RRID: SCR_012802) between samples. The output matrix of normalized counts was log-normalized and used as an input in the single scFEA software [30]. scFEA is a computational method, inferring fluxome from transcriptomics data. The output was composed of two matrices reporting the metabolites’ abundance and the reaction fluxes estimation, respectively, which were further merged into the clinical database.

### 4.3. Relation between One-Year eGFR and Each Metabolite Abundance Estimation

The relation between one-year eGFR and each metabolite abundance estimation as well as inferred reaction fluxes were fitted using a robust linear model. The ß parameter was estimated with the M-Estimator with Huber’s psi-function. The *p*-value was calculated through a robust F-Test, and a threshold of 0.05 was needed for significance.

For nonlinear fitting, we used a locally estimated scatterplot smoothing (LOESS) regression.

### 4.4. Random Forrest Model

We first assessed the pairwise correlation of every estimated metabolite. Fourteen pairs of metabolites exhibited a Pearson coefficient above 0.8. For each pair, we adopted three strategies:

- We only kept the final product (i.e., AMP, Dolichyl-phosphate-D-mannose, d-UTMP and Succinate).
- When two metabolites were correlated with a third one, we removed the third to maximize the number of predictors (Arginosuccinate, 3PD and Propanoyl-CoA).
- In the case of glutathione, glutamate and cysteine, we only kept glutathione, which has been associated with AKI or CKD in the literature [71,72,73].

The data were then randomly split in two sets, to train and test the model, respectively, in a 0.8:0.2 ratio. We used the matrix of metabolites’ estimated abundance, at the exclusion of the previously discarded variables, as a matrix of predictors for the RF model. Model optimization was performed using repeated K-fold cross-validation (5 repetitions of 10 K-fold cross-validations), which has recently been shown to outperform other resampling methods [74]. During this process, the model was tuned to find the optimal hyperparameters:

- The number of predictors randomly sampled as candidates for each split (mtry), were the square root and the log2 of the number of predictors, and a sequential vector of integer from 1 to the total number of predictors.
- The minimum sizes of terminal node (nodesize) tried were 2, 4 and 8.
- The numbers of trees to grow tried were 25, 50, 100, 150, 300, 500, 1000 and 2000.
- The optimal model was selected to maximize the RMSE.

The optimal model was finally applied to the test dataset, and the RMSE was calculated as a metric of accuracy. To estimate the standard deviation of the RMSE in the test dataset, we bootstrapped the test cohort 1000 times, applying the RF model on every resampling cohort and calculated the standard deviation.

### 4.5. Statistical Models

For the first model, we fitted the relation between the one-year eGFR (dependent variable) and the one-week serum creatinine (independent variable) with a linear regression, on the training dataset. We also used repeated K-fold cross-validation to assess the out-of-bag accuracy of the model in the training dataset. We further applied this model on the test dataset and calculated the RMSE with its standard deviation, as described in the previous paragraph.

For the second model, we first fitted the relation between the one-year eGFR (dependent variable) and each of the clinical and biological variables available. As three of these presented missing values (donor serum creatinine and age, hot ischemia time), they were imputed using bagged tree imputation, which has been shown to achieve the best imputation quality and improvements on the downstream predictions in a recent benchmark [36]. Variables with an invariable association with a one-year eGFR *p*-value below 0.3 were preselected. We thus used repeated K-fold cross-validation to find the optimal multivariable model, using a stepwise approach and a maximum number of predictors varying from 1 to the total number of available variables. The best model was selected to maximize the RMSE. This model was further tested on the test data, using the RMSE as the performance measure. The standard deviation of the RMSE metric in the test cohort was calculated as described in the previous paragraph.

### 4.6. TRIPOD Guidelines

To develop more reproducible approaches in the development of predictive models, a recent scientific initiative has suggested following standard guidelines [75]. For this purpose, the TRIPOD (Transparent reporting of a multivariable prediction model for individual prognosis or diagnosis) statement has been proposed. It consists of a 22-item checklist, which details vital information that must be incorporated into a prediction model study. In this study, we fulfilled these recommendations and have reported supporting information in the Supplementary Table S1.

## Figures and Tables

**Table 1:** Baseline characteristics, according to the median one-year eGFR.

	1-yr eGFR < 51 *n* = 21	1-yr eGFR > 51 *n* = 21	Total *n* = 42	*p*-Value
Donor serum creatinine (mg/dL)	0.8 (0.3)	0.7 (0.2)	0.7 (0.2)	0.180
Donor sex male	9 (42.9%)	10 (47.6%)	19 (45.2%)	1.000
Donor age (years)	39.3 (16.6)	55.9 (6.7)	47.8 (14.9)	0.002
Donor type				1.000
Donation after brain death	12 (57.1%)	13 (61.9%)	25 (59.5%)	
Deceased cadaveric donor	7 (33.3%)	6 (28.6%)	13 (31.0%)	
Living donor	2 (9.5%)	2 (9.5%)	4 (9.5%)	
Donor weight (kg)	71.5 (13.5)	77.0 (13.6)	74.2 (13.7)	0.175
Donor height (cm)	172.9 (8.6)	169.8 (8.6)	171.3 (8.6)	0.367
Donor body mass index (kg/m^2^)	23.9 (4.3)	26.7 (4.6)	25.3 (4.6)	0.002
Donor hypertension	1 (4.8%)	6 (28.6%)	7 (16.7%)	0.093
Recipient age (years)	50.0 (14.2)	54.6 (11.6)	52.3 (13.0)	0.385
Recipient sex male	14 (66.7%)	13 (61.9%)	27 (64.3%)	1.000
Cold ischemia time (hours)	11.4 (5.6)	12.9 (5.2)	12.2 (5.4)	0.187
Warm ischemia time (min)	49.5 (18.5)	47.9 (13.6)	48.7 (16.1)	0.995
Delayed graft function	2 (9.5%)	4 (19.0%)	6 (14.3%)	0.663
Immunosuppression				0.520
TAC-MMF-CS	9 (42.9%)	6 (28.6%)	15 (35.7%)	
Induction with Basiliximab	12 (57.1%)	15 (71.4%)	27 (64.3%)	
1-week Serum Creatinine (mg/dL)	2.8 (3.0)	4.0 (3.4)	3.4 (3.3)	0.110

**Table 2:** Training and test dataset characteristics, according to the median one-year eGFR.

	Cohort Test *n* = 8	Cohort Train *n* = 34	Total *n* = 42	*p*-Value
Donor serum creatinine (mg/dL)	0.8 (0.2)	0.7 (0.3)	0.7 (0.2)	0.352
Donor sex male	3 (37.5%)	16 (47.1%)	19 (45.2%)	0.760
Donor age (years)	40.7 (18.0)	49.4 (14.0)	47.8 (14.9)	0.227
Donor type				0.491
Donation after brain death	6 (75.0%)	19 (55.9%)	25 (59.5%)	
Deceased cadaveric donor	1 (12.5%)	12 (35.3%)	13 (31.0%)	
Living donor	1 (12.5%)	3 (8.8%)	4 (9.5%)	
Donor weight (kg)	71.0 (7.9)	75.0 (14.7)	74.2 (13.7)	0.508
Donor height (cm)	169.5 (10.0)	171.8 (8.4)	171.3 (8.6)	0.759
Donor Body Mass Index (kg/m^2^)	24.8 (2.6)	25.4 (5.0)	25.3 (4.6)	0.987
Donor hypertension	1 (12.5%)	6 (17.6%)	7 (16.7%)	0.093
Recipient age (years)	56.5 (10.7)	51.3 (13.5)	52.3 (13.0)	0.370
Recipient sex male	5 (62.5%)	22 (64.7%)	27 (64.3%)	1.000
Cold ischemia time (hours)	12.8 (5.6)	12.0 (5.4)	12.2 (5.4)	0.564
Warm ischemia time (min)	37.6 (8.5)	51.2 (16.4)	48.7 (16.1)	0.034
Delayed graft function	1 (12.5%)	5 (14.7%)	6 (14.3%)	1.000
Immunosuppression				0.425
TAC-MMF-CS	4 (50.0%)	23 (67.6%)	27 (64.3%)	
Induction with Basiliximab	4 (50.0%)	11 (32.4%)	15 (35.7%)	
1-week Serum Creatinine (mg/dL)	3.8 (3.8)	3.3 (3.2)	3.4 (3.3)	0.974
1-year Glomerular filtration rate (estimated, mL/min/1.73 m^2^)	57.4 (19.3)	52.2 (16.3)	53.2 (16.8)	0.586

**Table 3:** Accuracy metric of the three predictive models on the test dataset.

	RMSE	MAE	R2
random forest	12.2 ± 3.2	9.2 ± 2.9	0.5 ± 0.6
univariable linear model	24.4 ± 7.5	16.4 ± 6.68	−0.8 ± 1.0
stepwise linear model	23.2 ± 6.2	18.0 ± 5.4	−0.6 ± 1.0

## Data Availability

RNAseq data for human kidney biopsies are respectively available at GEO GSE126805.

## Supplementary Materials

The following are available online at https://www.mdpi.com/article/10.3390/metabo12010057/s1, Figure S1: Relation of the one-year eGFR with the estimation of metabolites abundance and reactions flux, Figure S2: data exploration, Figure S3: Random Forest model selection and Figure S4: Missing value imputation, Supplementary Table S1: TRIPOD guidelines report.
