# Supplementary material for: Estimated Renal Metabolomics at Reperfusion Predicts One-Year Kidney Graft Function
Source: Metabolites. 2022 Jan 10;12(1):57. doi: 10.3390/metabo12010057 (PMC8778290; doi:10.3390/metabo12010057)
Supplement: Supplementary file 1 [file metabolites-12-00057-s001.zip › metabolites-1523447-supplementary.pdf]

Supplementary Figure S1

a

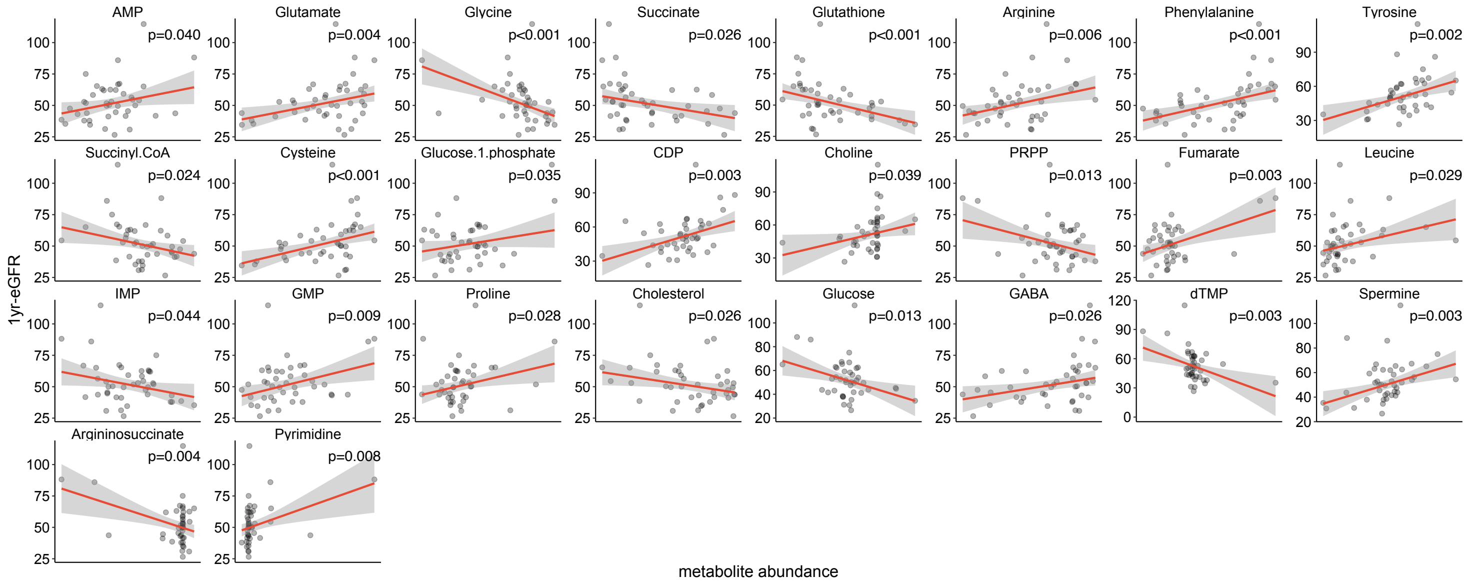

b

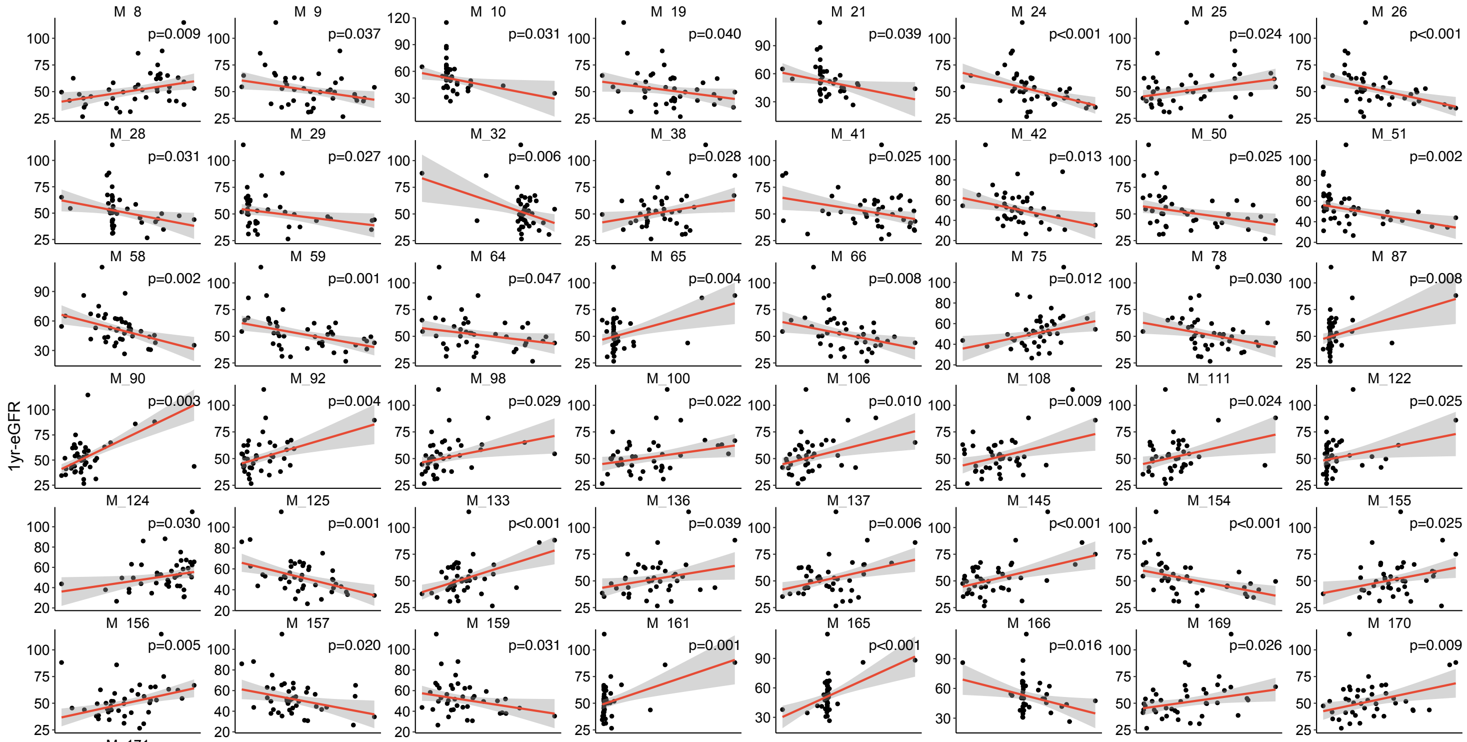

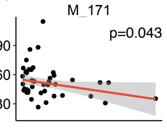

M_8	Citrate_2OG	M_92	Glutamine_in_Glutamine
M_9	2OG_Succinyl-CoA	M_98	Leucine_in_Leucine
M_10	Succinyl-CoA_Succinate	M_100	Arginine_Arginine
M_19	Glycine_Creatine	M_106	Glucose_Glucose-6-phosphate
M_21	Choline_Glycine	M_108	Glucose-6-phosphate_Glucose-1-phosphate
M_24	Glutathione_Glycine + Cysteine	M_111	Glucose-1-phosphate_Glycogen
M_25	Glutathione_glutamate	M_122	(GlcNAc)4 (Man)3 (Asn)1_(Gal)2 (GlcNAc)4 (LFuc)1 (Man)3 (Neu5Ac)2 (Asn)1
M_26	Glutamate + Cysteine_Glutathione	M_124	(GlcNAc)4 (Man)3 (Asn)1_(GlcNAc)5 (Man)3 (Asn)1
M_28	Serine_Methionine	M_125	Dolichyl phosphate D-mannose+Protein serine_(Gal)1 (GlcNAc)1 (Man)1 (Ser/Thr)1
M_29	Cysteine_Pyruvate	M_133	PRPP+Glutamine_IMP
M_32	Threonine_Propanoyl-CoA	M_136	IMP_AMP
M_38	Aspartate_Asparagine	M_137	IMP_XMP
M_41	Aspartate_B-Alanine	M_145	XMP_Xanthine
M_42	Spermine_B-Alanine	M_154	Uracil_B-Alanine
M_50	GABA_Succinate	M_155	UTP_CDP
M_51	Glutamate_2OG	M_156	CDP_Cytidine
M_58	Tyrosine_Tyrosine-OUT	M_157	CDP_dCDP
M_59	Phenylalanine_Tyrosine	M_159	dCMP_Deoxycytidine
M_64	Arginine_Putrescine	M_161	dCDP_dCTP
M_65	Argininosuccinate_Arginine + Fumarate	M_165	dTMP_dTTP
M_66	Citruline + Aspartate_Argininosuccinate	M_166	Thymine_Succinyl-CoA
M_75	Succinate_in_Succinate	M_169	Cholesterol_ Steroid_hormone
M_78	Lactate_Lactate_out	M_170	XMP_GMP
M_87	Pyrimidine_in_Pyrimidine	M_171	dCDP_dCMP
M_90	Propanoyl-CoA_in_Propanoyl-CoA		

Relation of the one-year eGFR with the estimation of metabolites' abundance and reactions' flux a) scatter plot showing one-year eGFR according to every metabolite estimated level, fitted with a robust linear regression and b) scatter plot showing one-year eGFR according to every flux, fitted with a robust linear regression.

Supplementary Figure S2

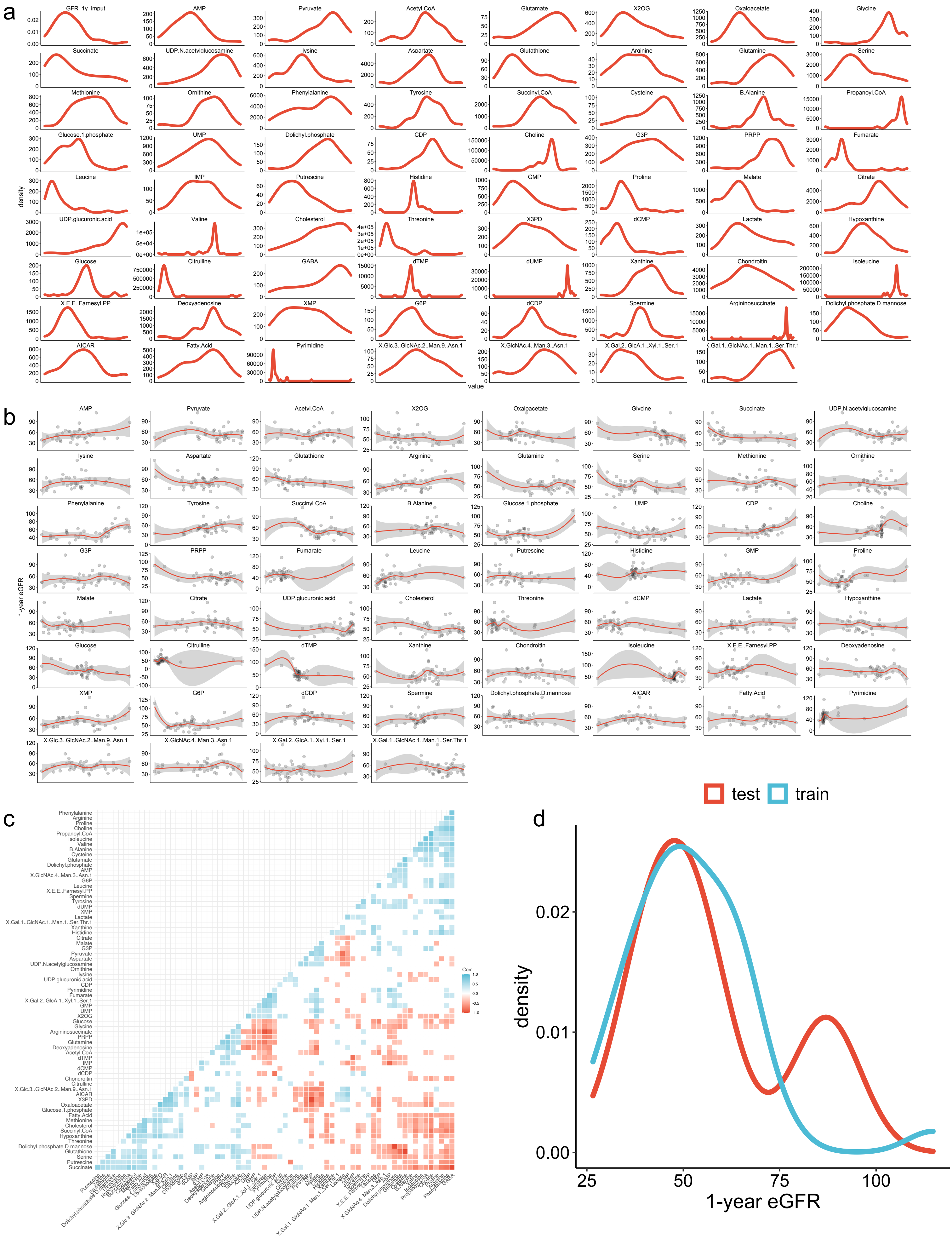

Data exploration: a) distribution of the estimations of metabolites' abundance, b) scatter plot showing one-year eGFR according to each metabolite estimation, fitted with non-linear (LOESS) function, c) correlation matrix for each couple of esimated metabolite abundance (red and blue colors indicate a negative and positive correlation respectively, blank cases refer to non significant correlations with a p-value>0.05) and d) distribution of one-year eGFR before the train and test datasets.

Supplementary Figure S3

a

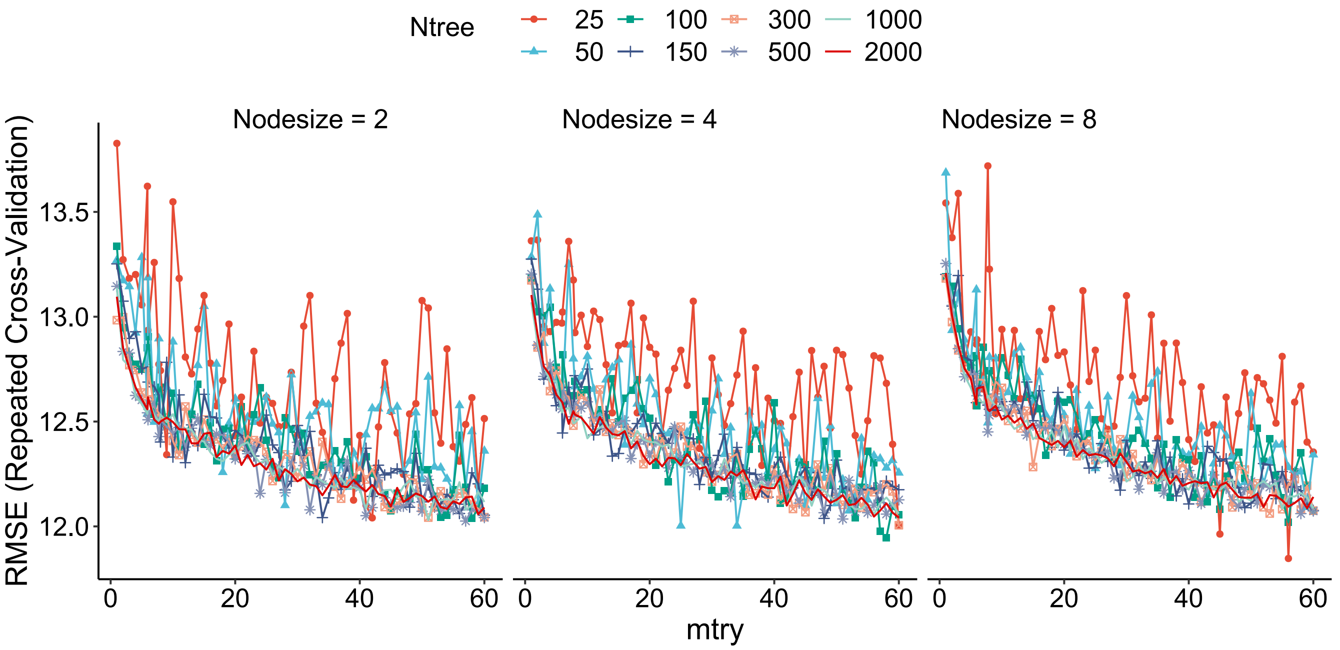

b

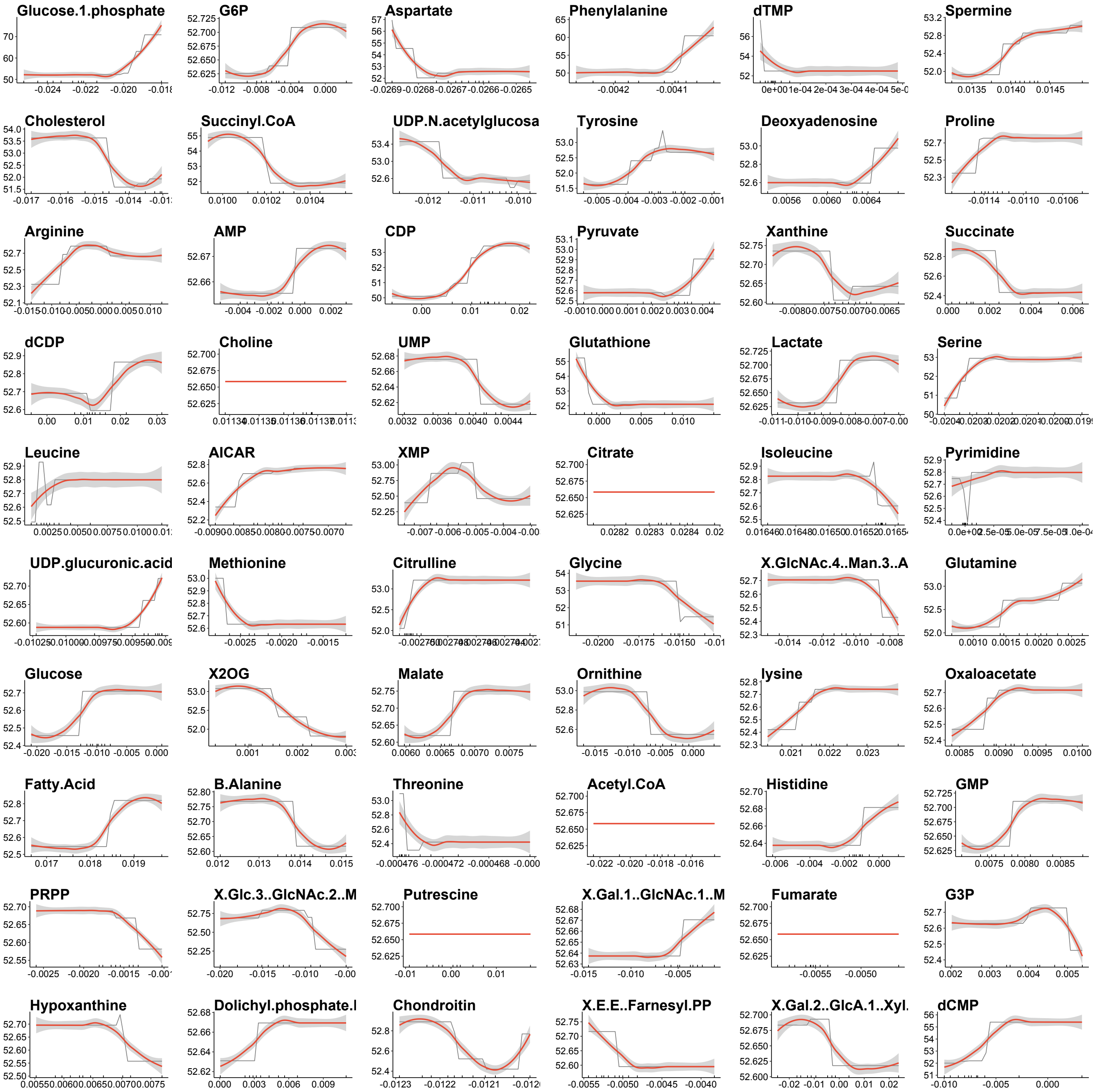

Random Forest model selection: a) Root Mean Square Error (RMSE) according to the number of predictors randomly sampled as candidate at each split (mtry), the number of trees to grow (ntee) and the the minimum size of terminal node (nodesize) and b) partial dependance plots showing the marginal effect of the variables on the predicted one-year eGFR.

Supplementary Figure S4

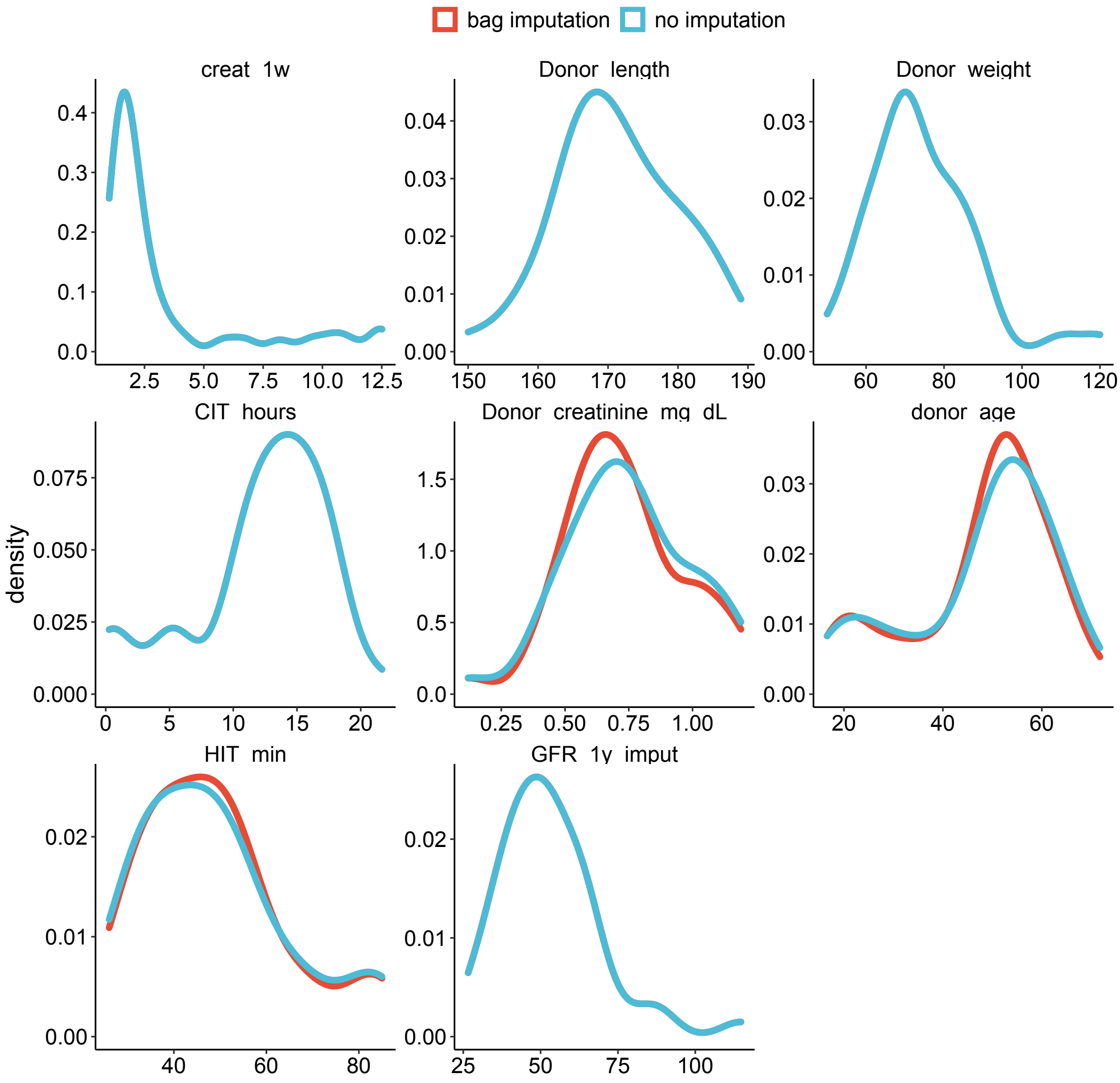

Missing values imputation: distribution of variables before (blue) and after (red) imputation of missing values

TRIPOD Checklist: Prediction Model Validation

Supplementary Table S1: TRIPOD guidelines report

Section/Topic	Item	Checklist Item	Section
**Title and abstract**			
Title	1	Identify the study as developing and/or validating a multivariable prediction model, the target population, and the outcome to be predicted.	See title.
Abstract	2	Provide a summary of objectives, study design, setting, participants, sample size, predictors, outcome, statistical analysis, results, and conclusions.	See abstract
**Introduction**			
Background and objectives	3a	Explain the medical context (including whether diagnostic or prognostic) and rationale for developing or validating the multivariable prediction model, including references to existing models.	Section 1
	3b	Specify the objectives, including whether the study describes the development or validation of the model or both.	Section 1
**Methods**			
Source of data	4a	Describe the study design or source of data (e.g., randomized trial, cohort, or registry data), separately for the development and validation data sets, if applicable.	Section 4, see [1]
	4b	Specify the key study dates, including start of accrual; end of accrual; and, if applicable, end of follow-up.	Section 4,see [1]
Participants	5a	Specify key elements of the study setting (e.g., primary care, secondary care, general population) including number and location of centres.	See [1]
	5b	Describe eligibility criteria for participants.	See [1]
	5c	Give details of treatments received, if relevant.	See [1]
Outcome	6a	Clearly define the outcome that is predicted by the prediction model, including how and when assessed.	Section 3
	6b	Report any actions to blind assessment of the outcome to be predicted.	N/A
Predictors	7a	Clearly define all predictors used in developing or validating the multivariable prediction model, including how and when they were measured.	Section 4
	7b	Report any actions to blind assessment of predictors for the outcome and other predictors.	N/A
Sample size	8	Explain how the study size was arrived at.	See [1]
Missing data	9	Describe how missing data were handled (e.g., complete-case analysis, single imputation, multiple imputation) with details of any imputation method.	Section 2
Statistical analysis methods	10c	For validation, describe how the predictions were calculated.	Section 4
	10d	Specify all measures used to assess model performance and, if relevant, to compare multiple models.	Section 2 and Section 4
	10e	Describe any model updating (e.g., recalibration) arising from the validation, if done.	N/A
Risk groups	11	Provide details on how risk groups were created, if done.	N/A
Development vs. validation	12	For validation, identify any differences from the development data in setting, eligibility criteria, outcome, and predictors.	Section 4
**Results**			
Participants	13a	Describe the flow of participants through the study, including the number of participants with and without the outcome and, if applicable, a summary of the follow-up time. A diagram may be helpful.	See [1]
	13b	Describe the characteristics of the participants (basic demographics, clinical features, available predictors), including the number of participants with missing data for predictors and outcome.	Section 2
	13c	For validation, show a comparison with the development data of the distribution of important variables (demographics, predictors and outcome).	Section 2
Model performance	16	Report performance measures (with CIs) for the prediction model.	Section 2
Model-updating	17	If done, report the results from any model updating (i.e., model specification, model performance).	N/A
**Discussion**			
Limitations	18	Discuss any limitations of the study (such as nonrepresentative sample, few events per predictor, missing data).	Section 3
Interpretation	19a	For validation, discuss the results with reference to performance in the development data, and any other validation data.	Section 3
	19b	Give an overall interpretation of the results, considering objectives, limitations, results from similar studies, and other relevant evidence.	Section 3
Implications	20	Discuss the potential clinical use of the model and implications for future research.	Section 3
**Other information**			
Supplementary information	21	Provide information about the availability of supplementary resources, such as study protocol, Web calculator, and data sets.	Section 4. supplementary materials.
Funding	22	Give the source of funding and the role of the funders for the present study.	Geneva University hospitals (HUG), Ernst and Lucie Schidheiny fundation

[1] Cippà, Pietro E., Bo Sun, Jing Liu, Liang Chen, Maarten Naesens, and Andrew P. McMahon. 'Transcriptional Trajectories of Human Kidney Injury Progression'. *JCI Insight* 3, no. 22 (15 November 2018): 123151. https://doi.org/10.1172/jci.insight.123151.
